# Kidney Transplantation for Erdheim-Chester Disease

**DOI:** 10.1155/2020/3954165

**Published:** 2020-07-13

**Authors:** Jongwon Yoo, Cynthia Gunsteen, Sima Patel, Ted Clevy-Schneller, Sucha Nand, Divya Jain, Raquel Garcia-Roca, Amishi Desai, Sanjeev Akkina

**Affiliations:** ^1^Department of Medicine, Transplant Nephrology, Loyola University Medical Center, Maywood, IL, USA; ^2^Department of Surgery, Intra-Abdominal Transplantation, Loyola University Medical Center, Maywood, IL, USA; ^3^Department of Hematology and Oncology, Loyola University Medical Center, Maywood, IL, USA

## Abstract

Erdheim-Chester disease is a rare inflammatory disease that infiltrates skeletal and extra-skeletal tissue. Chronic kidney disease (CKD) in Erdheim-Chester disease is usually attributed to retroperitoneal lesions that lead to urologic obstruction and hydronephrosis. In this report, we describe a patient diagnosed with Erdheim-Chester disease who eventually developed end-stage kidney disease (ESKD). After complete remission of Erdheim-Chester disease by vemurafenib therapy and 2 years of hemodialysis, the patient underwent a deceased donor kidney transplantation with basiliximab induction and tacrolimus/mycophenolic acid maintenance. After conversion of mycophenolic acid to azathioprine due to cost, acute cellular rejection had occurred, and he was treated with steroid therapy. The patient remained in complete remission from Erdheim-Chester disease and dialysis-free 16 months after transplant. Kidney transplantation is another treatment option for those patients with Erdheim-Chester disease who suffer from renal failure in the setting of complete remission.

## 1. Introduction

Erdheim-Chester disease (ECD) is a rare inflammatory disorder characterized by the accumulation of histiocytes [1]. Of the two types of histiocytosis, namely, Langerhans and non-Langerhans cell histiocytosis [2], ECD presents with an infiltration of non-Langerhans cell histiocytes in multiple organs [3]. Histiocytic infiltration involves an inflammatory process that causes granulation and necrotic debris and eventually results in xanthoma cells. The xanthogranulomatous process in ECD compromises multiple organs including the kidneys [2]. Renal dysfunction of patients with ECD has been reported elsewhere. In a retrospective study, among 31 ECD patients in one single center, twenty-two patients had infiltration in the renal pelvis [4]. And for those who had peri-renal infiltration, seventeen patients had compromised ureter which led to hydronephrosis. And fifteen patients or 50% of ECD patients were found to have infiltration in the renal vascular peduncle. The majority of the compromised renal function was caused by an obstructed urinary tract system, attributable to fibrosis development in the retroperitoneal area during the xanthogranulomatous process [3].

The prognosis for ECD is poor, especially when multiple organs are involved in the xanthoma cell formation process. Steroids, interferon alpha, and immunosuppressants are the main treatment modalities; however, heart and pulmonary involvement is indeed life threatening. Interestingly, Hashimoto's [5] team reported successful bilateral deceased donor lung transplantation in an ECD patient without recurrence of ECD at the 5-year mark posttransplantation in a setting with standard immunosuppressive agents including tacrolimus, mycophenolic acid, and steroids. Therefore, for patients with ECD who have developed end-stage kidney disease (ESKD), kidney transplantation may be a viable treatment option. Herein, we present, to our knowledge, the first reported case of kidney transplantation to treat ESKD for the patient with ECD.

## 2. Case Presentation

**Figure 1:**
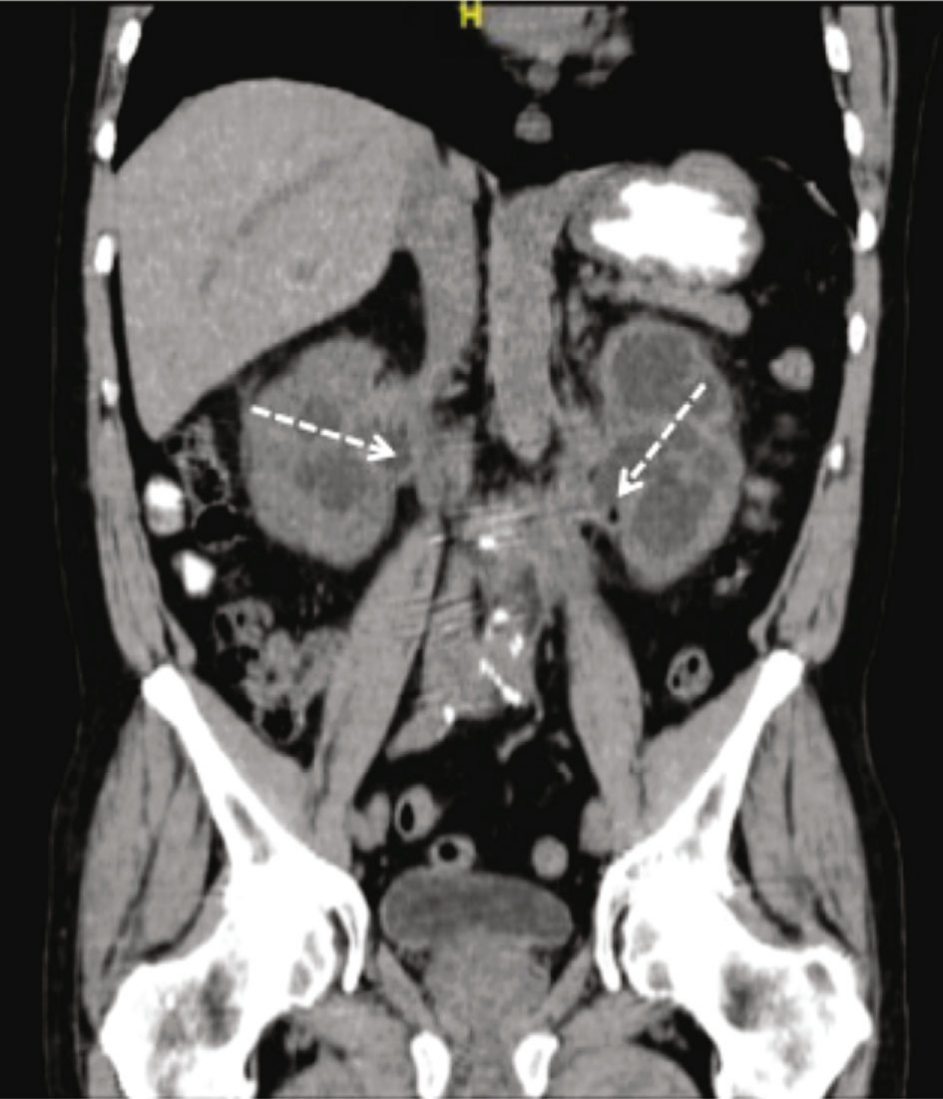
Computed tomography images of abdomen and pelvis of which dashed arrows indicated obstruction.

A 56-year-old male presented with uncontrolled hypertension and chronic kidney disease (CKD) with an estimated glomerular filtration of 17 ml/min/1.73m^2^ without proteinuria. Ultrasonography of his native kidneys indicated severe hydronephrosis with thinned renal cortex. The CT images indicated dilation of the right and left collecting systems with left renal cortical atrophy (Figure 1). The MAG3 renal scan indicated obstruction of the right kidney and a nonfunctioning left kidney. After ureteral stent placement, the patient suffered a prolonged hospitalization for candida pyelonephritis. During hospitalization, hypercalcemia was noted in spite of hypoparathyroidism. A bone survey was performed to rule out multiple myeloma, and it indicated bilateral distal femoral and proximal tibial sclerosis. The working diagnoses for sclerosis of bones were compromised vessels, osteomyelitis, neoplasm of bones, osteopoikilosis, Paget's disease, and Erdheim-Chester disease. Because of hypercalcemia, Paget disease was the most favored diagnosis. However, Erdheim-Chester disease could not be ruled out due to symmetric and long bone involved sclerosis. Therefore, a bone marrow biopsy was performed.

The bone biopsy showed histiocyte-rich bone marrow infiltration that was CD68 positive, that favored ECD given the multiple sclerotic lesions of the bones and other clinical manifestations including urological obstruction, hydronephrosis, and uncontrolled hypertension. The abnormal histiocytes showed BRAF V600E mutation and treatment with a BRAF inhibitor, vemurafenib 960 mg daily, was started with complete remission of ECD. Despite remission, renal function deteriorated, and hemodialysis was initiated 14 months after vemurafenib therapy and the dosages of vemurafenib were decreased to 480 mg twice a day in the setting of renal failure.

After 2 years of hemodialysis, the patient received a kidney allograft from a brain death donor with a kidney donor profile index of 91%. The kidney allograft was placed in the right iliac fossa without a ureteral stent. Basiliximab and methylprednisolone were used for induction therapy, and he was maintained on tacrolimus and mycophenolic acid after the transplant. Due to limited data in those with renal impairment, the dosage of vemurafenib remained unchanged at 480 mg twice a day, regardless of his eGFR throughout the posttransplant course. Due to cost, the patient was switched from mycophenolic acid to azathioprine and prednisone. Approximately 2 months posttransplantation, the serum creatinine level had risen to 2.38 mg/dl from nadir value of creatinine 1.93 mg/dl. Concurrently, the percentage value of donor derived cell free DNA (dd-cfDNA) from total cell free DNA [6] increased to 0.91% from a previous baseline of 0.60% measured at 1 month post transplantation. Because of the elevated creatinine and dd-cfDNA, he underwent a biopsy of the transplanted kidney. The pathology report indicated mild arteritis and c4d deposition in approximately 40% of the peritubular capillaries. Staining for polyoma virus was unremarkable. Given negative donor specific antibodies and mild arteritis, acute T cell-mediated rejection (Banff diagnostic category type IIA [7]) was diagnosed. This acute T cell-mediated rejection was likely associated with subtherapeutic immunosuppression at the time when converting mycophenolic acid to azathioprine. Methylprednisolone 500 mg daily for 3 days was administered intravenously, which was titrated down immediately to oral prednisone 5 mg daily. A subsequent biopsy after 1 month of therapy indicated c4d deposition of less than 10% and a low grade lymphocyte infiltration. During follow-up, dd-cfDNA decreased to 0.22%, *<*0.19%, and eventually *<*0.15% which was lower than that of median value (0.21%) found in transplant population with stable graft function [6]. One year eGFR was 48 ml/min/1.73 m^2^, and graft function remains stable 16 months after transplantation without any signs of urinary tract obstruction. With ongoing vemurafenib therapy, ECD has remained in complete remission. There are no signs of ECD involvement near the transplanted kidney and the ureteral system.

## 3. Discussion

After the two cases described by Jakob Erdheim and William Chester in 1930, the third case with similar clinical manifestations in 1970 was named Erdheim-Chester disease after the two physicians [8]. This histiocytic disorder is very rare. According to a comprehensive literature review [9], a total of 341 manuscripts written in English had described ECD patients up to June 2012. Among those manuscripts, two hundred fifty-nine patients had histologically proven ECD. And as of 2019, a total of 571 patients across the world are registered to the ECD Global Alliance with more than 50% of these or 238 patients being registered in the United States [10]. Aside from bone involvement, the ECD involvement in the urologic system is very common. For example, among 47 patients diagnosed with ECD at Mayo Clinic between 1996 and 2012, a total of 37 patients exhibited infiltration in retroperitoneum, hydronephrosis, or chronic kidney disease [11]. Renal diseases in ECD are attributable to the infiltration of histiocytes in the retroperitoneum, resulting in renal parenchyma compression and renovascular hypertension, obstruction of the urinary system, and hydronephrosis [12]. Relieving renal artery pressure via vascular stents or managing hydronephrosis with ureteral stents or percutaneous nephrostomy is the more likely symptom relief, while various medications target the pathogenesis of ECD (inflammation; IL-1; TNF alpha) and BRAF. Inhibition of the BRAF-V600E mutation with vemurafenib has been shown to improve ECD by clinical symptoms and laboratory/imaging [13] and is the only drug approved by the U.S. Food and Drug Administration [14]. Despite this therapy, for those ECD patients who develop ESKD eventually, dialysis therapy is a known treatment option.

However, we report here that kidney transplantation can be another treatment option for those with ECD who have failed kidneys. Solid organ transplantation in an ECD patient was previously reported. For one ECD patient with pulmonary fibrosis, bilateral lung transplantation from a deceased donor was performed successfully [5]. While there is an increased risk of recurrence in Langerhans cell histiocytosis in BRAF-V600E mutation [15], the lower risk of ECD recurrence in the patients with negative expression of BRAF-V600E enabled Hashimoto's team to predict favorable transplant outcomes. However, in this particular case report, despite having BRAF-V600E mutation, the patient has remained in remission of ECD with vemurafenib therapy. Vemurafenib therapy is shown to have a favorable metabolic response for those ECD patients with BRAF-V600E mutation [16], whereas a therapeutic strategy such as MEK inhibitors for the ECD patients without BRAF-V600E mutation has just started to emerge [17]. Even though concurrent therapy by sirolimus and prednisone is not the first treatment option for those who have ECD without BRAF-600E mutation, their efficacy on stabilizing ECD progression [18] warrants further study in those who undergo kidney transplantation.

In conclusion, kidney transplantation is another treatment option for those patients with ECD who develop ESKD in the setting of complete ECD remission. The standard induction and maintenance immunosuppressant regimen, including tacrolimus, mycophenolic acid, and prednisone, may be a reasonable strategy to protect the kidney allograft from rejection. Care by multidisciplinary teams, including hematology, is critical to assure there is complete remission at the time of transplantation and also to assess any ECD involvement posttransplantation.
